# Improving Lipid Production of *Yarrowia lipolytica* by the Aldehyde Dehydrogenase-Mediated Furfural Detoxification

**DOI:** 10.3390/ijms23094761

**Published:** 2022-04-26

**Authors:** Jiwon Kim, Hyeoncheol Francis Son, Sungmin Hwang, Gyeongtaek Gong, Ja Kyong Ko, Youngsoon Um, Sung Ok Han, Sun-Mi Lee

**Affiliations:** 1 Clean Energy Research Center, Korea Institute of Science and Technology (KIST), Seoul 02792, Korea; 216502@kist.re.kr (J.K.); hfrancis7@kist.re.kr (H.F.S.); sungminhwang@kist.re.kr (S.H.); gtgong@kist.re.kr (G.G.); jkko@kist.re.kr (J.K.K.); yum@kist.re.kr (Y.U.); 2 Department of Biotechnology, Korea University, Seoul 02841, Korea; samhan@korea.ac.kr; 3 Division of Energy and Environment, University of Science and Technology, Daejeon 34113, Korea; 4 KU-KIST Green School (Graduate School of Energy and Environment), Korea University, Seoul 02841, Korea

**Keywords:** *Yarrowia lipolytica*, lipids, furfural, tolerance, aldehyde dehydrogenase, YALI0E15400p

## Abstract

*Yarrowia lipolytica*, the non-conventional yeast capable of high lipogenesis, is a microbial chassis for producing lipid-based biofuels and chemicals from renewable resources such as lignocellulosic biomass. However, the low tolerance of *Y. lipolytica* against furfural, a major inhibitory furan aldehyde derived from the pretreatment processes of lignocellulosic biomass, has restricted the efficient conversion of lignocellulosic hydrolysates. In this study, the furfural tolerance of *Y. lipolytica* has been improved by supporting its endogenous detoxification mechanism. Specifically, the endogenous genes encoding the aldehyde dehydrogenase family proteins were overexpressed in *Y. lipolytica* to support the conversion of furfural to furoic acid. Among them, YALI0E15400p (FALDH2) has shown the highest conversion rate of furfural to furoic acid and resulted in two-fold increased cell growth and lipid production in the presence of 0.4 g/L of furfural. To our knowledge, this is the first report to identify the native furfural detoxification mechanism and increase furfural resistance through rational engineering in *Y. lipolytica*. Overall, these results will improve the potential of *Y. lipolytica* to produce lipids and other value-added chemicals from a carbon-neutral feedstock of lignocellulosic biomass.

## 1. Introduction

With the usage of fossil fuel encountering political and public opposition, the development of renewable energy sources is vital for environmental sustainability [1]. Accordingly, studies to find alternative manufacturing methods for renewable energy have arisen, spotlighting the biological route of using microorganisms as cell factories [2,3]. Lignocellulosic biomass, such as agricultural and forestry residue, serves as a renewable feedstock for microbial cell factories due to its low price and abundant availability [4]. However, the recalcitrance of lignocellulosic biomass requires a pretreatment process prior to microbial fermentation, from which fermentable sugars are generated along with various inhibitory compounds [5]. The presence of furan derivatives, such as 5-hydroxymethyl-2-furaldehyde (HMF) and 2-furaldehyde (furfural), hampers the microbial conversion of lignocellulosic biomass into fuels and chemicals. Specifically, furfural leads to the diminished conversion of lignocellulosic biomass into products by hampering the function of the enzyme involved in glycolysis and by accumulating reactive oxygen species (ROS) in the cell [6,7]. The decrease in the intracellular ATP and NAD (P)H by furfural also delays cell growth showing a prolonged lag phase [8].

Recently, various strategies for understanding and enhancing furfural tolerance have been applied to develop microbial cell factories for lignocellulosic biorefinery. In a model yeast of *Saccharomyces cerevisiae*, rational engineering by the overexpression of alcohol dehydrogenases (ADHs) or transcription factors, such as HAA1 and/or TYE7, increased furfural tolerance during xylose fermentation, in which strain performance is severely reduced by the presence of lignocellulosic biomass-derived inhibitors [9,10]. Adaptive laboratory evolution (ALE) has also improved furfural tolerance without prior knowledge and provided rational engineering targets [11]. *S. cerevisiae* with increased furfural tolerance was obtained by subculturing in a medium containing 60% (*v*/*v*) non-detoxified hydrolysate liquor for 100 generations [12]. The investigations of altered membrane permeability and ROS concentration during the detoxification of furfural have provided insight for developing engineering strategies to improve furfural tolerance [13,14]. In addition, intracellular redox perturbation using the enzyme overexpression-related interconversion of NADPH and NADP^+^, such as glucose-6-phosphate dehydrogenase (ZWF1) and glutathione oxidoreductase (GLR1), could increase furfural tolerance [15]. Isolated furfural tolerant strains also serve as essential sources to understand and manipulate furfural tolerance in yeast cell factories. *S. cerevisiae* strains tolerating three g/L of furfural have been identified by investigating a collection of over 70 environmental and industrial isolates [16]. Heterozygous intraspecies hybrid diploid strains of *S. cerevisiae* have been developed by crossing two isolate strains of Yllc17_E5α and UWOPS87-2421α, a high ethanol producer and a strain resistant against the inhibitors in lignocellulosic hydrolysates, respectively, providing robust strains for lignocellulosic ethanol production [17]. In non-model organisms, similar approaches have been applied to improve the perspectives of lignocellulosic biorefinery. In a filamentous fungus of *Neurospora crassa*, the transcriptomic analysis revealed the genes involved in furfural tolerance and the correlation between carbohydrate metabolism and furfural tolerance [18]. Beyond improving furfural tolerance, the selective conversion of furfural into furoic acid has been suggested as a promising bio-based upgrading strategy to generate value-added products from lignocellulosic biomass as reported in *Pseudomonas putida* [19] and *Nocardia coralline* [20].

*Yarrowia lipolytica* is a non-model oleaginous yeast that has recently emerged as one of the most promising production chassis for biofuel and oleochemicals. Its native lipid-production capacity has been increased to reach a lipid content of over 90% through engineering the lipid metabolism [21,22]. The easy accessibility of metabolic engineering tools, including the CRISPR-Cas9 system [23,24] and machine learning-based modeling [25], accelerates the developments of *Y. lipolytica* strains to become a powerful microbial cell factory. Recent reports on the use of lignocellulosic biomass as feedstock add more potential to *Y. lipolytica* for sustainable bioproduction [26]. However, the low tolerance of *Y. lipolytica* against inhibitory compounds in lignocellulosic hydrolysates limits the efficient conversion of lignocellulosic biomass into desired products. Specifically, furfural was found to be the most potent inhibitor on the growth of *Y. lipolytica* [27]. The growth inhibition was profound even in the absence of other inhibitory compounds [28]. The inhibitory effect was observed as the decreased lipid production during the bioreactor operation feeding pretreated lignocellulosic biomass as a substrate [26]. Despite severe inhibition, understanding the detoxifying mechanism and engineering efforts to enhance furfural tolerance have been less studied in *Y. lipolytica*. Adapting the engineering approaches used in a model yeast *S. cerevisiae*, the overexpression of alcohol dehydrogenase (*Sc*ADH6p), turned out to be ineffective in *Y. lipolytica* to obtain robust cell growth in using lignocellulosic biomass [29]. Though the recent report on controlling inoculum size provides a helpful solution to reduce the toxic effect of furfural [28], a more effective strategy to improve furfural tolerance would be required, based on the detoxification mechanism.

In this study, furfural tolerance was improved by investigating the detoxification mechanism in *Y. lipolytica*. The genes encoding aldehyde dehydrogenases (ALDHs) were overexpressed to enhance furfural tolerance, leading to improved cell growth and lipid production. The results contributed to the understanding of the furfural response mechanism, implying that ALDHs play critical roles in the response to furfural in *Y. lipolytica*. Hence, this study improves the potential of *Y. lipolytica* as an industrial workhorse to efficiently use lignocellulosic biomass as sustainable feedstock.

## 2. Results

### 2.1. Inhibitory Effect of Furfural on the Growth of Y. lipolytica

**Figure 1:**
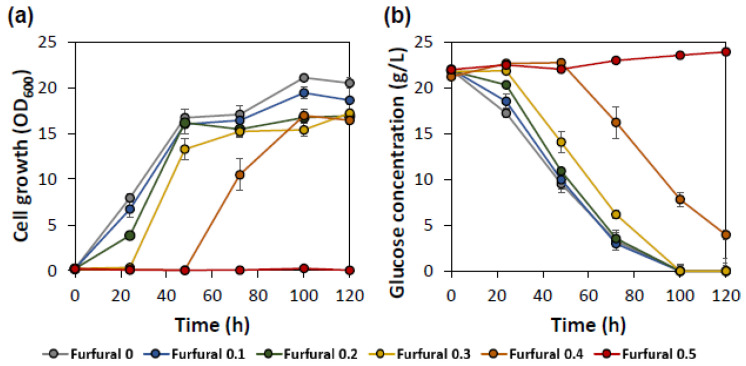
The inhibitory effect of furfural on the cell growth (**a**) and sugar consumption (**b**) of *Y. lipolytica*. Furfural was added to the CSM media at various concentrations of furfural (0–0.5 g/L). Error bars represent the standard deviation of biological triplicates.

Previous studies have shown that 0.5 g/L furfural could be lethal to *Y. lipolytica* cells [27], but furfural is often detected at concentrations below 0.5 g/L in non-detoxified lignocellulosic hydrolysates [30,31]. To determine the effect of furfural on *Y. lipolytica*, cell growth was monitored with various initial concentrations below 0.5 g/L. As shown in Figure 1, furfural inhibited the growth of *Y. lipolytica* resulting in the reduced OD, especially at the early stage of cultivation. At 24 h of cultivation, the OD of *Y. lipolytica* was decreased by almost half, even with 0.2 g/L of furfural (Figure 1). With the furfural concentration above 0.3 g/L, *Y. lipolytica* showed an extended lag phase, and no growth was detected with 0.5 g/L of furfural during 100 h of incubation, consistent with the previous study [27]. Of the tested conditions, the cells exhibited the most severe inhibition with 0.4 g/L of furfural. Thus, further experiments were conducted under the condition of 0.4 g/L furfural to investigate the engineering approaches to improve furfural tolerance in *Y. lipolytica*.

### 2.2. Elucidating a Furfural Detoxification Mechanism in Y. lipolytica

**Figure 2:**
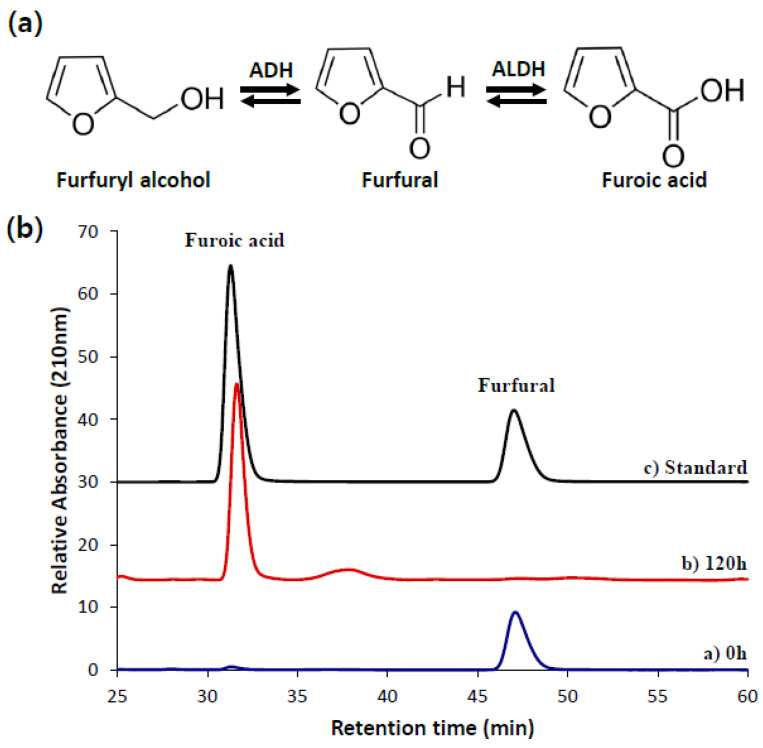
The hypothesized mechanism (**a**) of furfural detoxification in *Y. lipolytica* by either ADH or the ALDH converting furfural into furfuryl alcohol or furoic acid, respectively. The HPLC chromatograms (**b**) of the sample from the control strain expressing empty plasmid at the initial (0 h) and 120 h of incubation under a furfural stress condition.

Next, we tested a detoxification strategy from a model yeast of *S. cerevisiae* to determine whether the furfural tolerance is enhanced in *Y. lipolytica*. In *S. cerevisiae*, overexpressing the enzymes converting a reactive aldehyde of furfural to a less-toxic furfuryl alcohol, an alcohol dehydrogenase (ADH, EC 1.1.1.1) *Sc*ADH7p, and an aldehyde reductase (AHR, EC 1.1.1.2) *Sc*OSIp (YKL071wp), showed a positive result on relieving furfural inhibition [32,33,34]. To this end, we overexpressed *Sc*ADH7p and *Sc*OSI1p under the control of TEF promoter with UAS1B enhancer [35] and evaluated the inhibitory effect of furfural on the cell growth and sugar consumption of *Y. lipolytica*. However, the overexpression did not successfully restore cell growth and glucose consumption. The strains overexpressing *Sc*ADH7p and *Sc*OSI1p even showed reduced cell growth and glucose consumption compared to the control strain harboring an empty plasmid (Figure S1b,c). Unexpectedly, furoic acid was detected during the HPLC analysis of the culture product. After 120 h of cultivation, a furoic acid peak appeared as a furfural peak vanished (Figure 2b). A furfuryl alcohol peak was not detected throughout the cultivation for 170 h.

**Figure 3:**
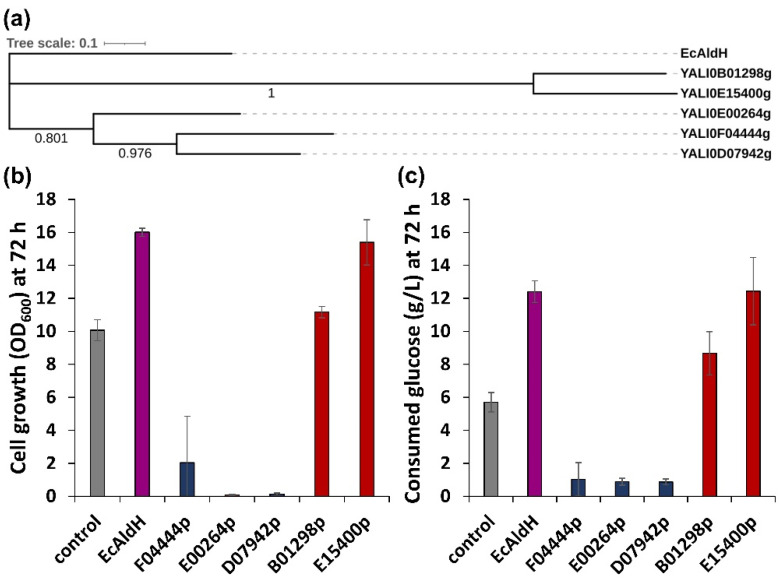
Comparison of the sequence similarities of the endogenous ALDHs, and the effect on the furfural tolerance in *Y. lipolytica*. (**a**) Phylogenetic tree of aldehyde dehydrogenases used in this experiment. Cell growth (**b**) and glucose consumption (**c**) of *Y. lipolytica* overexpressing various aldehyde dehydrogenases in the presence of 0.4 g/L furfural.

Based on the detection of furoic acid, we hypothesized that endogenous aldehyde dehydrogenase (ALDH, EC 1.2.1.3) plays a role in converting furfural to furoic acid, a lesser inhibitory compound in *Y. lipolytica* (Figure 2a). Hence, we conducted an overexpression of endogenous ALDH to improve furfural tolerance in *Y. lipolytica*. To this end, we selected five ALDH candidates, YALI0D07942p, YALI0E00264p, YALI0F04444p, YALI0E15400p, and YALI0B01298p, from Genbank using BLASTP search against an ALDH from *Escherichia coli* (*Ec*AldH) (Table 1). The overexpression of *Ec*AldH has been previously confirmed to relieve oxidative stress effectively in *Y. lipolytica* [36]. With a broad substrate range [36], we expected *Ec*AldH to convert furfural to furoic acid effectively. *Ec*AldH overexpression resulted in 1.6-fold increased cell growth measured by OD at 72 h and shortened a lag phase from 72 h to 24 h (data not shown), supporting our hypothesis that the overexpression of ALDH accelerates furfural conversion (Figure 3). Interestingly, YALI0E15400p, which showed the least similarity to *Ec*AldH, was shown to be the most effective in reducing the inhibitory effect of furfural in *Y. lipolytica* followed by YALI0B01298p. The overexpression of ALDHs with high similarity to *Ec*AldH, YALI0D07942p, YALI0E00264p, YALI0F04444p, were not effective in converting furfural into furoic acid, possibly due to improper conformation or poor expression of the enzymes.

### 2.3. Structural Analysis of the Effective ALDHs by a Homology Modeling and Docking Simulation

**Figure 4:**
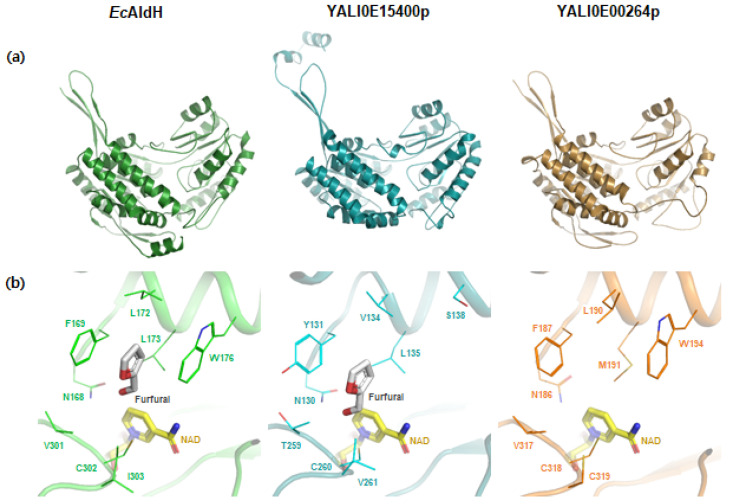
Monomeric structure (**a**) and docking simulation (**b**) of *Ec*AldH and endogenous ALDHs. Model structure and docking simulation of *Ec*AldH (left), YALI0E15400p (middle), and YALI0E00264p (right). The core residues forming a substrate-binding pocket are shown as a line model and labeled appropriately. The furfural and NAD ligands are shown as stick models with grey and yellow colors, respectively.

To discover a mismatch between the sequence similarity and the detoxification performance of ALDHs, we employed a computational homology modeling approach and molecular docking simulation on two endogenous ALDHs, YALI0E15400p and YALI0E00264p, with low and high similarities to *Ec*AlDH, respectively (Figure 4). The homology modeling analysis revealed that three model structures have canonical ALDH conformation and *Ec*AldH and YALI0E00264p have almost identical structures. A molecular docking simulation of furfural into ALDH structures showed that eight residues were mainly involved in forming a furfural-binding pocket in the *Ec*AldH and YALI0E15400p with high affinity of furfural (−4.8 and −4.0 kcal/mol, respectively). In *Ec*AldH, six residues, Phe169, Leu172, Leu173, Trp176, Val301, and Ile303, contributed to hydrophobic cavity formation for furfural binding. In particular, *Ec*AldH^Phe169^ and *Ec*AldH^Asn168^ residues were mainly involved in stabilizing the furfural located near the catalytic residue of *Ec*AldH^Cys302^. YALI0E15400p has a similar substrate-binding cavity for furfural, in which polar Asn residue forming a hydrogen bond with aldehyde group and catalytic Cys residue are completely conserved as YALI0E15400p^Asn130^ and YALI0E15400p^Cys260^, respectively. YALI0E15400p^Tyr131^ residue is located at the corresponding site of *Ec*AldH^Phe169^, and contributes to stabilizing the furan ring of furfural by hydrophobic pi-pi stacking interaction. These observations indicated that various residues in each enzyme formed a suitable hydrophobic cavity for furfural stabilization. YALI0E00264p with higher sequence similarity also has conserved Asn and Cys residues as YALI0E00264p^Asn186^ and YALI0E00264p^Cys318^, respectively, and various hydrophobic residues for the furfural binding cavity formation. However, the YALI0E00264p^Met191^ residue appears to interfere with furfural stabilization, and a polar YALI0E00264p^Cys319^ residue appears to have a negative effect on hydrophobic pocket formation and, thus, stabilizes furfural to a lesser degree.

### 2.4. Improving Furfural Tolerance by the Overexpression of Fatty Aldehyde Dehydrogenases in Y. lipolytica

**Figure 5:**
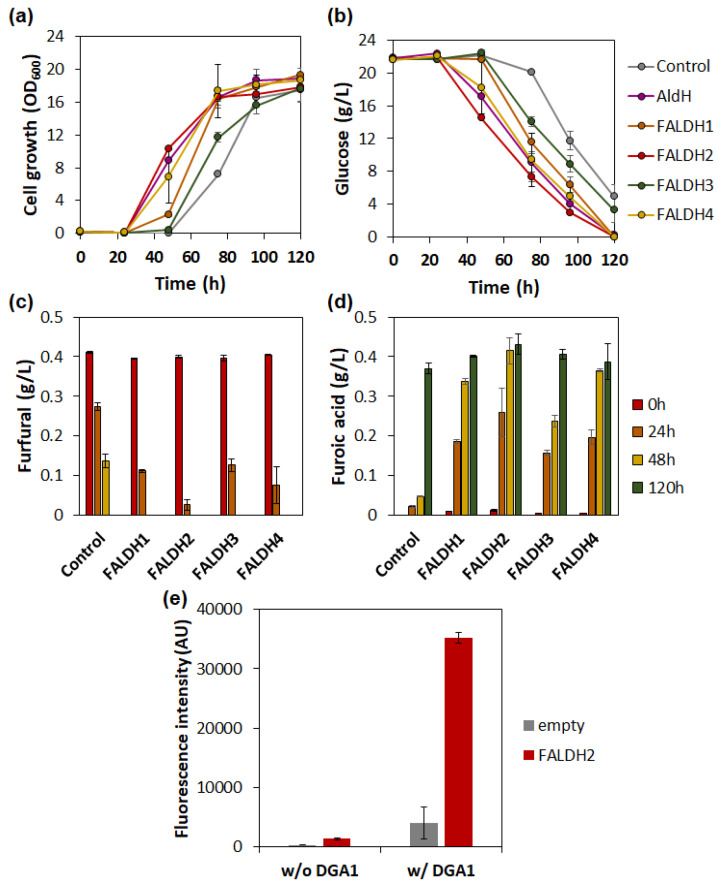
Effect of FALDH overexpression on the cell growth and lipid production of *Y. lipolytica* in the presence of 0.4 g/L furfural. Cell growth (**a**), glucose consumption (**b**), and the conversion of furfural (**c**) into furoic acid (**d**) of *Y. lipolytica* expressing various fatty aldehyde dehydrogenases. Lipid production (**e**) was measured by using a (Nile-red assay in the *Y. lipolytica* expressing FALDH2 and FALDH2/DGA1 at 96 h of incubation. Error bars represent the standard deviation of biological triplicates.

The ALDHs with functional furfural aldehyde dehydrogenase activity, YALI0E15400p and YALI0B01298p, were reported to be FALDH2 and FALDH3 [37,38]. In *Y. lipolytica*, four genes encoding FALDHs are present, which are often involved in n-alkane metabolism [38] (Table S5), opening up the possibility of identifying other enzymes effective in the conversion of furfural to furoic acid. Therefore, we overexpressed two additional FALDHs in *Y. lipolytica* along with the previously conformed FALDH2 and FALDH3. The overexpression of FALDHs showed an improved conversion of furfural to furoic acid and cell growth compared to the control strain harboring empty plasmids under the furfural stress condition (Figure 5a). FALDH2 was found to be the most effective in converting furfural to furoic acid, resulting in an over two-fold increased growth rate compared to the control (0.09 vs. 0.22 h^−1^). Some 93% of furfural was utilized within 24 h of incubation, and the complete conversion of furfural into furoic acid was observed at 48 h in the strain expressing FALDH2 (Figure 5d). Finally, we confirmed the effect of increased furfural tolerance on the lipid production of *Y. lipolytica*. The strain expressing FALDH2 produced 2.6-fold higher lipid measured by Nile-red staining than the control strain expressing empty plasmids (Figure 5e), indicating that lipid synthesis was accelerated under furfural stress conditions. The improvement was more profoundly observed in the strain with higher lipid production capacity (Figure 5e). When FALDH2 was co-expressed with diacylglycerol acyltransferase (DGA1), a common overexpression target for high lipid production in *Y. lipolytica* [39], nine-fold higher fluorescence was measured compared to the control strain.

## 3. Discussion

Inhibitory compounds derived from lignocellulosic biomass constrain the cellular growth and production performance of microbial cell factories. Furfural is a primary inhibitor that severely affects the cell performance of *Y. lipolytica*. Previously, engineering approaches to improve furfural tolerance were adapted from a model yeast of *S. cerevisiae* by overexpressing alcohol or acetaldehyde reductases to convert furfural to less toxic furfuryl alcohol [40,41]. However, the expression of *Sc*ADH7p [32] and *Sc*OSI1p [33] was not effective in improving the furfural tolerance of *Y. lipolytica*, implying there would be a difference in the furfural detoxification mechanism between *S. cerevisiae* and *Y. lipolytica*. *S. cerevisiae*, often growing in an anaerobic condition, exclusively converts furfural to furfuryl alcohol [42]. As proposed in this study, an obligate aerobic yeast of *Y. lipolytica* [43] seems to evolve to overcome furfural inhibition using an alternative route, the conversion of furfural to furoic acid. This detoxification mechanism is found in some aerobic bacteria such as *Pseudomonas putida* and *E. coli* [44]. Oleaginous yeasts of *Trichosporon cutaneum* and *Trichosporon fermentans* also convert furfural into furoic acids [45,46]. However, furfuryl alcohol is often detected as an intermediate at a particular time point when culturing these yeasts. In *T. cutaneum*, furfuryl alcohol was detected only at the beginning of the cultivation, leaving furoic acid as a single product of furfural degradation for the rest of the time [45]. On the other hand, the presence of furfuryl alcohol lasted over 200 h during the cultivation of *T. fermentans* [46]. This opens up the possibility of the conversion of furfural into furfuryl alcohol followed by fast conversion into furoic acid, which could have led to no furfuryl alcohol detection during the culture of *Y. lipolytica*. Nevertheless, the conversion of furfural into furoic acid seems to be the dominant detoxification mechanism in *Y. lipolytica* since the overexpression of alcohol dehydrogenases from *S. cerevisiae* did not improve furfural tolerance. Interestingly, the amount of furoic acid produced was almost the same as the amount of furfural added in the medium. No further conversion of furoic acid was observed during 120 h of cultivation. These suggest that no catabolic pathway for furoic acid utilization exists in other oleaginous yeasts, such as *Trichosporon cutaneum* [45]. Given that the selective biosynthesis of furoic acid is gaining interest as an upgrading lignocellulosic biomass [19,20], co-production of lipids and furoic acid would further improve the potential of *Y. lipolytica* as a workhorse for lignocellulosic biorefinery.

The enzymes in the FALDH superfamily tend to convert furfural to furoic acid more efficiently than endogenous ALDHs. Of the FALDHs, FALDH2 was the most effective in converting furfural to furoic acid, followed by FALDH4 and FALDH1 (Figure 5a). FALDHs overexpression shortened the lag phase of the cell under the furfural stress condition, implying the rate of furfural conversion defines the initial growth and thus final lipid production in *Y. lipolytica* during aerobic fermentation.

The efficiency of FADHs in converting furfural to furoic acid seems to be determined by the structure of the enzymes, in which the residues forming a hydrophobic cavity play an important role. Through homology modeling, we predicted the structure of the FADHs and *Ec*AldH to understand the discrepancy between the sequence similarity and the detoxification efficiency. In four FALDHs with furfural dehydrogenase activity, canonical ALDH conformation was confirmed, in which Cys residues for catalysis and Asn residues for hydrogen bond formations are completely conserved. In addition, a Tyr residue-stabilizing furan ring by pi-pi interaction was also conserved in all FALDHs. Among the five hydrophobic residues in FALDH2, three (Tyr131, Leu135, and Val261) are completely conserved in all FALDHs. These conserved residues seem to confer furfural aldehyde dehydrogenase activity while unconserved hydrophobic residues determine the furfural utilization capacity differences (Figure S2).

## 4. Materials and Methods

### 4.1. Strains and Culture Conditions

*Yarrowia lipolytica* PO1f strains (ATCC MYA-2613) were used in this study. The yeast strain was grown in a yeast synthetic complete (YSC) medium with 50 mM potassium phosphate buffer at pH 6.8, which contained 6.7 g/L yeast nitrogen base (YNB), 20 g/L glucose, and a complete supplement mixture (CSM) or CSM-Leu or CSM-Leu-Ura (MP Biomedicals, Solon, OH, USA). To evaluate furfural resistance, *Y. lipolytica* was inoculated into 100 mL flasks containing 20 mL of the corresponding medium with furfural 0–0.5 g/L at an initial OD600 of 0.2 and cultured at 28 °C with constant shaking at 200 rpm. *E. coli* DH10β (New England BioLabs, Ipswich, MA, USA) was used for cloning and plasmid propagation. *E. coli* DH10β cells were grown at 37 °C in Luria–Bertani medium supplemented with 100 µg/mL of ampicillin (Sigma Aldrich, St. Louis, MO, USA) with constant shaking at 200 rpm.

### 4.2. Plasmid and Strain Construction

All plasmids and strains used in this study are summarized in supplementary Tables S1 and S2. To construct a plasmid expressing aldehyde dehydrogenase and other genes (*Sc*ADH7p and *Sc*OSI1p), genomic DNA was extracted from *E. coli* DH10β, *Y. lipolytica* PO1f, and *S. cerevisiae* BY4741 using Wizard Genomic DNA Purification Kit (Promega, Madison, WI, USA). Gene fragments were amplified from appropriate genomic DNA through polymerase chain reaction (PCR) with primers including AscI/PacI enzyme sites as an overhang. After purification, all DNA fragments were ligated into pMCS plasmid with UAS1B enhancer and TEF promoter [35] using AscI and PacI restriction enzyme. The constructed plasmids were confirmed by enzyme digestion and Sanger sequencing and transformed into *Y. lipolytica* using a Frozen EZ Yeast Transformation II Kit (Zymo Research, Irvine, CA, USA).

### 4.3. Phylogenetic Analysis

An amino acid sequence of aldehyde dehydrogenase (ALDH) from *E. coli* (RefSeq No. WP_001009090.1) was queried to investigate orthologs in the genome of *Y. lipolytica* by using BlastP from the BLAST package [47]. A total of 13 ALDHs were predicted, followed by extracting the specific sequences of aldehyde dehydrogenase domain (IPR015590) based on the Interpro database [48]. The trimmed sequences were aligned using MUSCLE [49] in the MEGA 11 platform [50]. The resulting alignments were used for a phylogenetic tree construction by the maximum-likelihood inference with the JTT model in the MEGA 11. For the estimation of confidence for the tree topology, 1000 bootstrap replications were applied. The tree was visualized by the iTOL environment [51].

### 4.4. Protein Homology Modeling and Molecular Docking Simulation

The protein model structures of *Ec*AldH (Uniprot P23883), FALDH2 (YALI0E15400p), YALI0E00264p were built using a protein structure homology modeling server, SWISS-MODEL [52,53]. PDB codes of template structures, amino acid sequence identity between query and template sequences, and QMEANDisCo global scores are shown in Table S3. TM-scores were calculated with pairwise structure alignment in protein data bank (PDB) web service [54,55]. The model structures of *Ec*AldH, FALDH2, and YALI0E00264p were compared using jFATCAT (rigid) parameters [56,57].

Molecular docking simulation of furfural to three aldehyde dehydrogenase (ALDH) structures was performed by using AutoDock Vina [58]. Three ALDH model structures, *Ec*AldH, FALDH2, YALI0E00264p, were superimposed to chain A of *Ec*AldH model structure, and then docking simulation was performed. Marvin was used for drawing the furfural chemical structure to molecular docking simulation [59]. The pdbqt files were generated by AutoDock Tools, and all steps were performed by the AutoDock Vina manual [60]. The grid center and size information are shown in Table S4. Out of 90 docking poses generated from the docking simulation of furfural to *Ec*AldH and FALDH2, the one with the most appropriate direction and distance between the aldehyde group of furfural and the catalytic residue was selected. For YALI0E00264p, no suitable docking pose was obtained even with the simulation of 180 docking poses, of which furfural chemical structures were not properly located at the substrate-binding pocket.

### 4.5. Lipid Analysis

To estimate lipids produced by *Y. lipolytica*, the Nile-red assay was performed according to the previous study [61] with modifications: In brief, 100 µL of culture samples grown for 96 h were harvested by centrifugation for 3 min at 10,000× *g* and resuspended in 500 µL PBS buffer (pH 6.8). The samples were stained by adding 10 µL of 1 mM Nile-red (Sigma Aldrich, St. Louis, MO, USA) solution in DMSO and dark-incubated for 15 min at 30 °C. After centrifuging and washing with ice-cold water, the stained samples’ fluorescence signals were measured using TECAN Infinite Pro 200 (Tecan Group Ltd., Männedorf, Switzerland) equipped with excitation and emission filters for 535 nm and 580 nm wavelength, respectively.

### 4.6. Analytical Method

To estimate yeast cell growth, optical density (OD) of the culture broth was measured using a spectrophotometer UV-1240 (Shimadzu, Kyoto, Japan). The glucose, furfural, and furoic acid concentrations were quantified using a high-performance liquid chromatography system (HPLC, Agilent Technology 1100 series) equipped with refractive index detectors with an Aminex HPX-87H column (Bio-Rad Inc., Hercules, CA, USA). The mobile phase was 5 mM H_2_SO_4_ with a flow rate of 0.6 mL/min, and the column temperature was kept at 50 °C. Before analysis, each sample was filtered by a 0.22-µm syringe filter (Whatman, Kent, UK).

## 5. Conclusions

Enhancing furfural tolerance is a practical engineering strategy to improve the potential of *Y. lipolytica* as a production host for lignocellulosic biorefinery. Here, we investigated the furfural detoxification mechanism in *Y. lipolytica* and applied the knowledge to improve furfural tolerance through rational engineering. The overexpression of FALDH effectively alleviated cellular toxicity and accelerated the conversion of sugars into lipids. Thus, this study provides new insight into the effective bioconversion of lignocellulosic biomass containing furfural as an inhibitory compound and offers an effective engineering strategy to improve the potential of *Y. lipolytica* as a production host for lignocellulosic biorefinery.

## Figures and Tables

**Table 1:** Description of the aldehyde dehydrogenases used in this study.

Enzyme	Annotation	Cofactor	Similarity to *Ec*AldH	Growth Improvement *
*Ec*AldH	Aldehyde dehydrogenase	NAD^+^	-	Yes
YALI0F04444p	YER073w-like aldehyde dehydrogenase	NADP^+^	40%	No
YALI0E00264p	YOR374w-like aldehyde dehydrogenase	NAD^+^	40%	No
YALI0D07942p	YMR170c-like aldehyde dehydrogenase	NAD^+^	40%	No
YALI0B01298p	Fatty aldehyde dehydrogenase 3	NAD^+^	28.5%	Yes
YALI0E15400p	Fatty aldehyde dehydrogenase 2	NAD^+^	28.5%	Yes

* Data adopted from Figure 3; the cell growth of *Y. lipolytica* in the presence of 0.4 g/L furfural.

## Data Availability

Data presented in the article or Supplementary Materials.

## Supplementary Materials

The following supporting information can be downloaded at: https://www.mdpi.com/article/10.3390/ijms23094761/s1.
