# Supplementary material for: Improving Lipid Production of Yarrowia lipolytica by the Aldehyde Dehydrogenase-Mediated Furfural Detoxification
Source: Int J Mol Sci. 2022 Apr 26;23(9):4761. doi: 10.3390/ijms23094761 (PMC9102794; doi:10.3390/ijms23094761)
Supplement: Supplementary file 1 [file ijms-23-04761-s001.zip › ijms-1679676-supplementary.pdf]

International Journal of *Molecular Sciences*

MDPI

Supplementary Material

Table S1. List of plasmids used in this study.

Plasmid	Expression Cassette	Reference
pMCS-empty	Empty plasmid with leu marker	Blazeck et al. 2011
pMCS-ScADH7p	UAS1B16-TEF(504)p-ScADH7p-CYC1t	This study
pMCS-ScOSI1p	UAS1B16-TEF(504)p-ScOSI1p-CYC1t	This study
pMCS-EcAldH	UAS1B16-TEF(504)p-EcAldH-CYC1t	This study
pMCS-F04444p	UAS1B16-TEF(504)p-F04444p-CYC1t	This study
pMCS-E00264p	UAS1B16-TEF(504)p-E00264p-CYC1t	This study
pMCS-D07942p	UAS1B16-TEF(504)p-D07942p-CYC1t	This study
pMCS-FALDH1	UAS1B16-TEF(504)p-FALDH1-CYC1t	This study
pMCS-FALDH2	UAS1B16-TEF(504)p-FALDH2-CYC1t	This study
pMCS-FALDH3	UAS1B16-TEF(504)p-FALDH3-CYC1t	This study
pMCS-FALDH4	UAS1B16-TEF(504)p-FALDH4-CYC1t	This study
pMCSu-DGA1	UAS1B16-TEF(504)p-DGA1-CYC1t	This study

Table S2. List of primers used in this study.

Primer	Sequence	Reference
ScADH7-F	ACGTCAGGCGCGCCATGCTTTACCCAGAAAAATTTCAGG	This study
ScADH7-R	GCTGACTTAATTAACTATTTATGGAATTTCTTATCATAATCGACCAAAG	This study
ScOSI1-F	ACGTCAGGCGCGCCATGAATACTTCATCAAGAA-TAACTTACTTTATCATCGGT	This study
ScOSI1-R	GCTGACTTAATTAATTATCTAAAAGACGCCTTCGCTGCCG	This study
EcAldH-F	ACGTCAGGCGCGCCATGAATTTTCATCATCTGGCTTACTGG	This study
EcAldH-R	GCTGACTTAATTAATCAGGCCTCCAGGCTTATC	This study
F04444-F	ACGTCAGGCGCGCCATGCCATATATACGGTGTCTGGG	This study
F04444-R	GCTGACTTAATTAACTACAACTTCATACCCATGTTAATATGCAC	This study
E00264-F	GTCAGGCGCGCCATGCTCCGACGAATCACTCT	This study
E00264-R	GACTTAATTAATCACTGCTTGTCAGCCCA	This study
D07942-F	GTCAGGCGCGCCATGCAAGTTACTCTTCCCGACG	This study
D07942-R	GACTTAATTAACTAATCCAGGTTAATGTGGACGG	This study
FALDH1-F	GTCAGGCGCGCCATGTCCTGGGAAACAATCACTC	This study
FALDH1-R	GACTTAATTAACTACTTAATAAACACCGACATAATCTGAG	This study
FALDH2-F	GTCAGGCGCGCCATGTCAGAGTTCGATTGGGAGT	This study
FALDH2-R	GACTTAATTAATCATAAGAAAATTCCTGACTTCAAACTGG	This study
FALDH3-F	TCAGGCGCGCCATGACTACCACTGCCACAGAGAC	This study
FALDH3-R	ACTTAATTAACTAGTTGAAGAGTCTCGACCAAAAT	This study
FALDH4-F	GTCAGGCGCGCCATGTCTACCTTTGATTGGGAATCCA	This study
FALDH4-R	GACTTAATTAACTAGAGCAGAGCCTTGGC	This study
DGA1-F	ACGTCAGGCGCGCCATGACTATCGACTCACAATACTACAAGTC	This study
DGA1-R	TAGCGATTAATTAACTTACTCAATCATTCGGAACTCTGG	This study

Table S3. PDB templates and the estimated quality of protein homology modeling.

	Template PDB Code	Sequence Identity	QMEANDisCo Global	TM value (*Ec*AldH)
*Ec*AldH	5IUV	57.99%	0.85 ± 0.05	-
FALDH2 (YALI0E15400)	4QGK	39.78%	0.75 ± 0.05	0.80
YALI0E00264	5FHZ	50.94%	0.80 ± 0.05	0.93

Table S4. The grid center and size for molecular docking simulation.

	Grid Center (x, y, z)	Grid Size (x, y, z)
*Ec*AldH	−53.805, 14.115, 181.243	42, 46, 38
FALDH2 (YALI0E15400)	−53.805, 14.115, 181.243	42, 46, 38
YALI0E00264	−53.805, 15.805, 181.243	46, 58, 40

Table S5. Annotation of fatty aldehyde dehydrogenases (FADLHs) in *Y. lipolytica.*

Gene	Locus
FALDH1	YALI0A17875p
FALDH2	YALI0E15400p
FALDH3	YALI0B01298p
FALDH4	YALI0F23793p

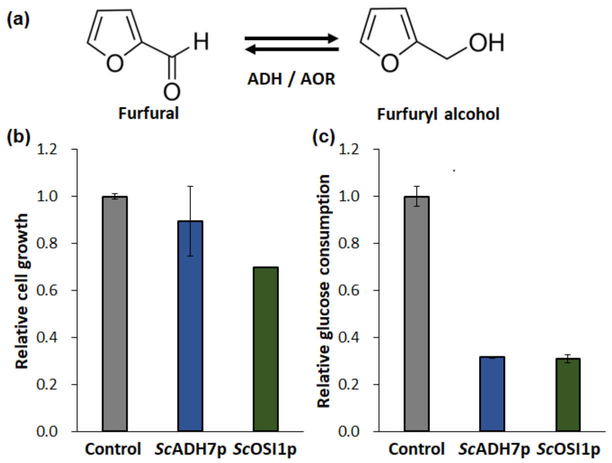

Figure S1. Effect of the overexpression of furfuryl alcohol-converting enzymes (*Sc*ADH7 and *Sc*OSI1) on the cell growth and the glucose consumption of *Y. lipolytica* under furfural stress (0.4 g/L). (**a**) Oxidoreduction between furfural and furfuryl alcohol by alcohol-converting enzymes, (**b**,**c**) the relative cell growth (b) and the glucose consumption (**c**) of *Y. lipolytica* expressing *Sc*ADH7 or *Sc*OSI1, an alcohol dehydrogenase from *S. cerevisiae*, compared to the strain expressing an empty plasmid at 170 h of incubation. The cell growth was measured by OD_{600nm}. Error bars represent the standard deviation of biological triplicates. ADH, alcohol dehydrogenase; AOR, aldehyde oxidoreductase.

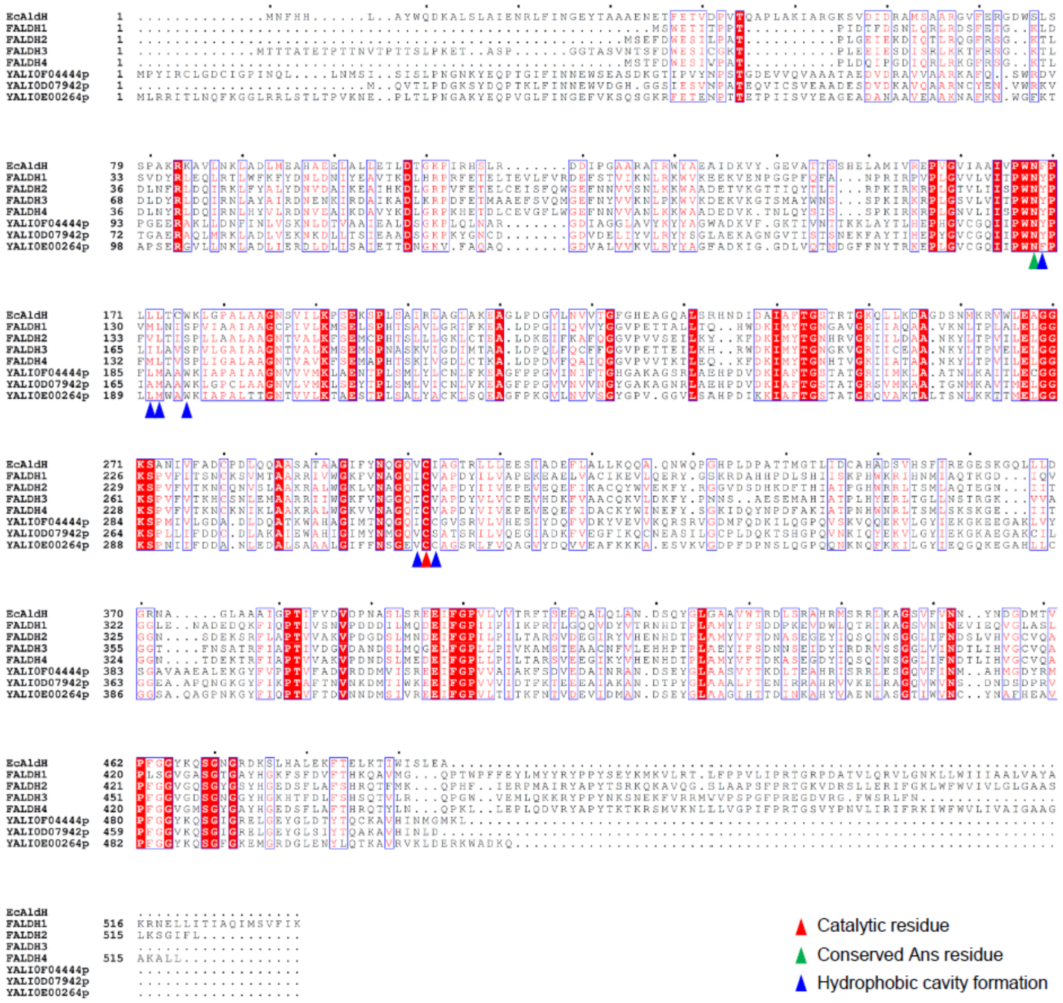

Figure S2. Sequence alignment of *Ec*AldH and endogenous aldehyde dehydrogenases.
